# Comparing doctors’ legal compliance across three Australian states for decisions whether to withhold or withdraw life-sustaining medical treatment: does different law lead to different decisions?

**DOI:** 10.1186/s12904-017-0249-1

**Published:** 2017-11-28

**Authors:** Ben P. White, Lindy Willmott, Colleen Cartwright, Malcolm Parker, Gail Williams, Juliet Davis

**Affiliations:** 1 Australian Centre for Health Law Research, Faculty of Law, Queensland University of Technology, Brisbane, Australia; 2 Southern Cross University, Gold Coast, Australia; 3 Faculty of Medicine, University of Queensland, Brisbane, Australia; 4 School of Public Health, University of Queensland, Brisbane, Australia

**Keywords:** End-of-life decision-making, Medical law, Withholding and withdrawing life-sustaining treatment, Compliance with law, Advance directives

## Abstract

**Background:**

Law purports to regulate end-of-life care but its role in decision-making by doctors is not clear. This paper, which is part of a three-year study into the role of law in medical practice at the end of life, investigates whether law affects doctors’ decision-making. In particular, it considers whether the fact that the law differs across Australia’s three largest states – New South Wales (NSW), Victoria and Queensland – leads to doctors making different decisions about withholding and withdrawing life-sustaining treatment from adults who lack capacity.

**Methods:**

A cross-sectional postal survey of the seven specialties most likely to be involved in end-of-life care in the acute setting was conducted between 18 July 2012 and 31 January 2013. The sample comprised all medical specialists in emergency medicine, geriatric medicine, intensive care, medical oncology, palliative medicine, renal medicine and respiratory medicine on the AMPCo Direct database in those three Australian states. The survey measured medical specialists’ level of legal compliance, and reasons for their decisions, concerning the withholding or withdrawal of life-sustaining treatment. Multivariable logistic regression was used to examine predictors of legal compliance. Linear regression was used to examine associations between the decision about life-sustaining treatment and the relevance of factors involved in making these decisions, as well as state differences in these associations.

**Results:**

Response rate was 32% (867/2702). A majority of respondents in each state said that they would provide treatment in a hypothetical scenario, despite an advance directive refusing it: 72% in NSW and Queensland; 63% in Victoria. After applying differences in state law, 72% of Queensland doctors answered in accordance with local law, compared with 37% in Victoria and 28% in NSW (*p* < 0.001). Doctors reported broadly the same decision-making approach despite differences in local law.

**Conclusions:**

Law appears to play a limited role in medical decision-making at the end of life with doctors prioritising patient-related clinical and ethical considerations. Different legal frameworks in the three states examined did not lead to different decisions about providing treatment. More education is needed about law and its role in this area, particularly where law is inconsistent with traditional practice.

**Electronic supplementary material:**

The online version of this article (10.1186/s12904-017-0249-1) contains supplementary material, which is available to authorized users.

## Background

While the proper role for law in medical practice is contested [1], there is no doubt that law plays an increasingly significant role in regulating medicine. This includes end-of-life care, with most countries having legal frameworks governing decisions about life-sustaining treatment [2–6]. These laws generally recognise, for example, the power to appoint substitute or surrogate decision-makers and to complete advance directives. As such, doctors have important legal responsibilities in this area including: assessing the patient’s capacity to consent to the withholding or withdrawing of treatment, identifying an authorised decision-maker where a patient lacks capacity, and, importantly for this paper, whether an advance directive needs to be followed.

Previous studies suggest that the role of advance directives in doctors’ end-of-life decision-making is fraught [7–10]. For example, Burkle et al. [8] suggest that doctors’ compliance with advance directives is “situation-specific” (p. 5), with medical judgment superseding advance directives where clinically indicated. This is supported by findings that many intensive care doctors believe end-of-life decisions to be primarily medical ones, even where there is an advance directive [9]. In addition to clinical factors, previous research has also found that doctors identified ethical considerations to be more determinative of their actions than advance directives [7].

This paper examines compliance with the law at the end of life, based on a hypothetical survey scenario involving an advance directive. It is part of a three-year study which has considered the role of law in decisions to withhold or withdraw life-sustaining treatment from adults who lack capacity. Previous papers from this study have examined issues such as whether doctors know the law in this area [11], whether legally knowledgeable doctors are more likely to follow the law [12], and the role of law for palliative care specialists, including their part in advancing end-of-life legal knowledge as a trusted source of information for other doctors, nurses, patients and families [13]. The focus of the present paper is a natural experiment which tests the impact of critical differences in end-of-life law across three Australian jurisdictions: New South Wales (NSW), Victoria and Queensland. If law influences medical decision-making at the end of life, different results in different states would be expected to reflect their different law – but this was not the case.

These findings are relevant for comparing legal compliance across countries but are particularly important for federated nations, such as the United States, Canada and Australia, where the law in this area varies across the country by state or province. The challenges posed by this variation within a country’s laws have been recently lamented by DeMartino et al in the case of the United States [14]. This paper responds to DeMartino et al’s call for empirical research to understand properly whether and how variation in law can impact on medical decision-making for adults who lack capacity.

## Methods

This study used a postal survey to explore legal compliance among medical specialists involved in end-of-life decision-making, and the extent to which these specialists considered law to be relevant when making such decisions. The states of NSW, Victoria and Queensland were chosen because 77% of all doctors in Australia practise there [15] and because of important variation in the law of these states. The survey instrument was developed over 18 months and was informed by a detailed review of the law in each state [16–18], the accuracy of which was confirmed by independent legal experts. The instrument was refined through focus groups, pretesting, and piloting with doctors.

Following ethics approval, the survey was distributed to doctors whose specialties most often involved in end-of-life decisions, i.e. emergency medicine, geriatric medicine, intensive care, medical oncology, palliative medicine, renal medicine and respiratory medicine. These specialties were determined to be most likely to be involved in decisions about withholding and withdrawing life-sustaining treatment through a literature review, interviews and an analysis of pilot survey results.

Using AMPCo Direct (a subsidiary of the Australian Medical Association), surveys were sent to 2858 doctors resulting in a final sample of 2702 after excluding those not at the contact address or not currently or previously in the relevant specialty. AMPCo Direct has Australia’s most comprehensive and accurate doctor database and has been used in other major studies of Australian doctors [19].

AMPCo Direct administered the survey mail-out from July 2012. Strategies to improve response included having the survey instrument professionally designed, providing incentives (continuing professional development points, educational material, and a chance to win one of six bottles of prestige wine), engaging with the Colleges and Societies of target specialties, and publishing editorials in relevant professional journals where possible to request participation in the study [20, 21]. Two follow-up requests were sent to non-responders and the survey closed on 31 January 2013.

A more detailed description of the development of the survey instrument, and the wider project methodology, has been published elsewhere [22].

### Measures

The survey contained six sections: perspectives on the law; education and training on the law; knowledge of the law in the participant’s state; the participant’s practice and compliance with the law; experience in making end-of-life decisions; and demographics. The compliance section contained three questions which required participants to respond to a hypothetical scenario in which Mark, a 53-year-old man who had been diagnosed with AIDS five years earlier, presented at hospital in a delirious state with bacterial pneumonia. Mark had a valid advance directive (local legal terminology used), drafted soon after his AIDS diagnosis, which stated that if a life-threatening infection arose, he did not wish to receive antibiotics but only to be kept comfortable. His family provided the advance directive but insisted that he be treated for the infection. If given antibiotics, Mark was expected to fully recover from the pneumonia. If antibiotics were withheld, it was likely that Mark would die.

In the first question, participants selected whether they would or would not commence antibiotics and then chose a statement which they felt best described the reason for their decision. The second question allowed participants to explain further their answer if necessary. The third question put forward a number of factors relating to professional practice and ethics, personal views, legal compliance, patient-related factors, family views, and an “other” option (Table 3), which participants were asked to score based on relevance to their decision-making process, on a scale from 1 (not relevant) to 4 (very relevant).

### Statistical analysis

Questionnaires were coded and double-entered into an Access database and transferred to SPSS Statistics 20 (IBM) and SAS 9.4 (SAS Institute Inc.) for analysis. Preliminary analyses examined descriptive statistics and bivariate associations using chi-square tests. For this component of the study, multivariable logistic regression was used to examine predictors of compliance with the law. Linear regression was used to examine the associations between the decision to commence antibiotics and the relevance of factors to making this decision, as well as state differences in these associations. Post-hoc comparisons among states within treaters (doctors who would provide the antibiotics) and non-treaters (doctors who would not provide the antibiotics) were adjusted using the Bonferroni procedure. The Pearson chi-square test was used to compare reasons for treating or not treating, omitting one category with small numbers in each case.

## Results

The overall response rate was 32% (867/2702): 29% (335/1147) from NSW, 32% (314/957) from Victoria and 36% (218/598) from Queensland. The respondent sample was similar to the original AMPCo sample on most compared variables (age, gender, specialty, and state) except that there were fewer younger doctors among respondents than in the sample population (Additional file 1: Comparison of AMPCo database and study sample).

### Decisions to treat and compliance with law

A majority of specialists in each state would not follow the advance directive in the scenario but would commence antibiotics; respondents from NSW (72%) and Queensland (72%) were significantly more likely to do so than respondents from Victoria (63%) (*p* = 0.029) (Table 1). However, differences in law meant that, legally-speaking, the advance directive should be followed in NSW and Victoria but not in Queensland, where treatment should be given (Additional file 2: Explanation of Law). This means that 72% of Queensland specialists who responded to the survey complied with the law, compared with only 37% of Victorian specialists and 28% of New South Wales specialists (Table 1). Demographic variables were of limited utility in predicting compliance, perhaps reflecting the disparity in law across the three states.

**Table 1:** Decisions about Treatment and Level of Compliance with Law. Responses by State % (n)

		Commence antibiotics % (n)	Not commence antibiotics % (n)	Complied with law % (n)	Did not comply with law % (n)
NSW	329	72 (236)	28 (93)	28 (93)	72 (236)
Queensland	215	72 (155)	28 (60)	72 (155)	28 (60)
Victoria	309	63 (195)	37 (114)	37 (114)	63 (195)
Total^a^	853	69 (586)	31 (267)	42 (362)	58 (491)
		P = 0.029		p < 0.001	

^a^14 respondents did not nominate a state

### Reasons given for decision

Of those specialists who said they would commence antibiotics and not follow the advance directive, a significant majority (70% in NSW and Queensland and 73% in Victoria) selected the reason that “The advance directive is relevant to my decision-making process but other factors are more relevant” (Table 2). In Queensland, where providing treatment was the lawful response, only 3% of respondents gave the most legally-correct answer, i.e., that the advance directive did not have legal effect; another possible legal justification was that it was not necessary to follow the advance directive if doing so was not clinically indicated and 14% of respondents chose this reason. Therefore, just 17% of doctors complying with the law in Queensland identified reasons for their decision that reflected the law in that state.

**Table 2:** Reasons Given for Decision to Commence Antibiotics or Not. Responses by State % (n)

Reasons for commencing antibiotics (not following advance directive)	NSW % (n)	QLD % (n)	VIC % (n)
I do not have to follow the advance directive because it is inconsistent with what is clinically indicated	13 (31)	14 (21)	11 (21)
The advance directive is relevant to my decision-making process but other factors are more relevant	70 (164)	70 (108)	73 (124)
The advance directive is not relevant to my decision-making because I don’t believe advance directives are appropriate to determining treatment	2 (4)	1(1)	1 (1)
The advance directive does not have legal effect	3 (8)	3(5)	6 (12)
Other	10 (24)	10 (17)	8 (16)
Total	**100 (231)**	**100 (152)**	**100 (192)**
Reasons for not commencing antibiotics (following advance directive)	**NSW** **% (n)**	**QLD** **% (n)**	**VIC** **% (n)**
The most important consideration is following the patient’s wishes	43 (40)	33 (20)	30 (34)
The most important consideration is that the law requires me to follow the advance directive	8 (7)	10 (6)	4 (5)
Both of the above considerations are equally important	48 (45)	57 (34)	64 (73)
Other	0	0	2 (2)
Total	**100 (92)**	**100 (60)**	**100 (114)**

The majority of those who chose to not commence antibiotics considered the patient’s wishes and a perceived legal requirement to follow the advance directive to be of equal importance (Table 2). The only demographic variable that reached significance across either set of reasons was gender, and only in relation to reasons for following the advance directive and not treating (chi-square, 2df, *p* = 0.016). Female specialists were significantly more likely to say that their decision-making was equally dependent on the patient’s wishes and the law whereas male specialists were significantly more likely to say that the most important consideration in their decision-making was following the patient’s wishes. It is of note that, despite the very different law in Queensland, differences by state did not reach significance for either set of reasons.

### Factors relevant in decision-making

Participants ranked how relevant given factors were to their decision-making in the survey scenario (Table 3). Patient-oriented considerations such as “patient’s expected quality of life after proposed treatment” and “whether treatment is clinically indicated” were more important than issues of legal compliance such as “following the law” and “following the patient’s advance directive”. This was the case across the overall sample and all states.

**Table 3:** Relevance of Factors in Decision to Commence Antibiotics or Not. Responses by State and by Treating or Not Treating (Mean Score from 1 to 4 (SD)), in descending order of importance

Factor (Overall Mean)	1 NSW Treat	2 NSW Not Treat	3 QLD Treat	4 QLD Not Treat	5 VIC Treat	6 VIC Not Treat	7 TOTAL Treat	8 TOT AL Non-Treat
	NSW P value Treat vs Not Treat		QLD P value Treat vs Not Treat		VIC P value Treat vs Not Treat		TOTAL P value Treat vs Not Treat	
Patient’s expected quality of life after proposed treatment (3.43)	3.72 (0.57)	2.84 (0.95)	3.72 (0.51)	2.83 (0.94)	3.72 (0.53)	2.73 (0.98)	3.72 (0.54)	2.79 (0.95)
	**<0.001**		**<0.001**		**<0.001**		**<0.001**	
Whether treatment is clinically indicated (3.18)	3.41 (0.64)	2.73 (0.97)	3.48 (0.62)	2.61 (0.85)	3.45 (0.65)	2.53 (0.91)	3.44 (0.64)	2.61 (0.92)
	**<0.001**		**<0.001**		**<0.001**		**<0.001**	
Your personal ethical principles (2.95)	3.04 (0.87)	3.02 (0.92)	3.09 (0.84)	2.57 (1.13)	2.97 (0.90)	2.68 (1.06)	3.03 (0.87)	2.77 (1.04)
	0.89		**<0.001**		**0.008**		**<0.001**	
Following the patient’s advance directive (2.90)	2.52 (0.70)	3.68 (0.47)	2.63 (0.63)	3.52 (0.50)	2.57 (0.67)	3.65 (0.51)	2.57 (0.67)	3.63 (0.50)
	**<0.001**		**<0.001**		**<0.001**		**<0.001**	
Following the law (2.77)	2.58 (0.79)	3.07 (0.72)	2.65 (0.66)	3.10 (0.54)	2.62 (0.76)	3.16 (0.69)	2.61 (0.75)	3.11 (0.67)
	**<0.001**		**<0.001**		**<0.001**		**<0.001**	
Family views (2.70)	2.83 (0.74)	2.29 (0.73)	3.06 (0.70)	2.45 (0.77)	2.80 (0.68)	2.23 (0.78)	2.88 (0.72)	2.30 (0.76)
	**<0.001**		**<0.001**		**<0.001**		**<0.001**	
Professional guidelines (2.69)	2.68 (0.81)	2.70 (0.79)	2.69 (0.84)	2.68 (0.85)	2.67 (0.82)	2.75 (0.85)	2.68 (0.82)	2.72 (0.83)
	0.89		0.98		0.37		0.53	
Views of colleagues about what you should do (2.67)	2.66 (0.83)	2.49 (0.83)	2.90 (0.72)	2.70 (0.94)	2.81 (0.86)	2.23 (0.87)	2.77 (0.82)	2.43 (0.89)
	0.11		0.12		**<0.001**		**<0.001**	
Hospital policies (2.16)	2.08 (0.92)	2.30 (0.91)	1.99 (0.85)	2.23 (0.91)	2.18 (0.87)	2.28 (0.96)	2.09 (0.89)	2.28 (0.93)
	**0.045**		0.074		0.37		**0.008**	
Concerns about being sued or criminal prosecution (2.09)	1.95 (0.89)	2.22 (0.83)	2.11 (0.91)	2.28 (0.99)	2.08 (0.89)	2.12 (0.98)	2.04 (0.90)	2.19 (0.93)
	0.18		0.21		0.71		**0.022**	
Your religious views (1.25)	1.32 (0.70)	1.27 (0.67)	1.25 (0.64)	1.15 (0.44)	1.23 (0.53)	1.12 (0.43)	1.27 (0.63)	1.18 (0.53)
	0.52		0.26		0.16		0.068	

Unsurprisingly, the preferencing of patient-oriented considerations over legal compliance was also reflected when comparing those who would provide treatment despite the advance directive with those who would not treat. In all states, and overall, treaters scored significantly higher than non-treaters on quality of life considerations and whether the treatment was clinically indicated, and significantly lower than non-treaters on following the patient’s advance directive and the law. Treaters were also more likely to score “family views” higher than non-treaters.

Treaters from each state were also compared with each other (comparing columns 1, 3 and 5 of Table 3), as were the non-treaters from each state (comparing columns 2, 4 and 6). Despite the very different law in Queensland, there were only two significant differences in the weight assigned by treaters to the eleven decision factors across states: the views of family (*p* = 0.002) and colleagues (*p* = 0.016). Similarly, there were only two significant differences in how non-treaters across the three states weighted the eleven factors: “your personal ethical principles” (*p* = 0.015) and again “views of colleagues” (*p* = 0.003). (Data not shown in table.) It is noteworthy that even at this very granular level of weighting across a large range of decision-making factors, there is marked overlap between how treaters as a group decide and how non-treaters as a group decide – despite the law in one state being the opposite to that in the other two jurisdictions.

## Discussion

This study supports findings in other research that doctors are unlikely to comply with advance directives refusing treatment where clinical, and perhaps some ethical, considerations favour giving treatment [7–10]. This is reflected in the low level of compliance with the advance directive in the scenario as well as the reasons given for the decision to treat and the factors doctors ranked as relevant in their decision-making. These results also point to a hierarchy where law is less important than patient-oriented clinical factors in these decisions.

What is novel in these findings is the use of a natural experiment based on differing law. While harmonisation of advance care planning legislation within a nation is a long-standing policy objective in many jurisdictions [14, 23], variation in law across states or provinces presents an opportunity for natural experiments to test the impact of law on medical decision-making. In this study, Australian doctors were faced with a scenario in which the application of end-of-life law produced an outcome in NSW and Victoria which was the opposite of that in Queensland. Despite this legal variation, doctors in Queensland made broadly the same decisions as doctors in the other two states.

Queensland doctors also gave broadly the same *reasons* for their decisions (very few selecting legally correct options) as doctors from the other two states. This similarity in approach was particularly clear, especially in comparison with NSW, in relation to the reasons for providing treatment and not following the advance directive where clear differentiated choices for treating were available. It was less clear for those who chose to follow the advance directive and not provide treatment, which is likely, in part, to be due to a clumping of responses as there were only three overlapping choices for this option. But even then, these results did not reveal statistically significant variation despite Queensland’s different law. Further, when examining the *factors* that doctors rated as relevant in their decision-making, there were only limited differences between what Queensland doctors thought were relevant and what doctors from the other two states took into account.

These results suggest, at least in relation to the survey scenario, that the law is not an important influence on doctors’ end-of-life decision-making. If law were an important consideration, the variation in law between Queensland and NSW and Victoria should be reflected in doctors making different decisions, doing so for different reasons and with differently-weighted factors. Yet there were no significant differences in reasons and very few in terms of the weight given to factors in decision-making. Further qualitative research would be worthwhile to explore in more depth the role that law plays in these decisions, and indeed how the various clinical and non-clinical factors influence decision-making in this setting.

The limited impact of law is also evidenced in the global findings about compliance with law in the scenario. It was very low in NSW (28%) and only a little better in Victoria. In Queensland, compliance was much higher at 72% but arguably that is likely to be because of the clinical considerations pointing in that direction rather than the law. In other words, we question if this was intentional compliance with law. There is also the issue of 28% of Queensland doctors not complying with the law and following an advance directive which would lead to a person’s death. At least some of this cohort, we suggest, would be “accidental non-compliers”; mistakenly believing that they were following the law based on their incorrect understanding of it.

Part of this non-compliance could be due to a lack of knowledge. Elsewhere we have identified significant gaps in doctors’ legal knowledge [11] but increasing knowledge is not sufficient, in and of itself, to lead to compliance with law. It is also necessary for the role, utility and relevance of law to be understood [12] and acted upon. That said, increasing knowledge may have an effect on compliance where the law in question is counterintuitive and/or inconsistent with widely held views in the medical profession and with accepted ethical positions. The Queensland law in this case, where an advance directive does not have to be followed in situations such as those outlined in the scenario, is an example of such law.

This study also sheds light on a more modest natural experiment. Whereas the discussion above relates to the same or similar decisions despite very different law, it is also possible to see different decision-making despite the *same* law. Victoria and NSW have the same legal result for this scenario yet compliance was significantly higher in Victoria. It is possible that specialists in Victoria are more inclined to law, perhaps because of different training and education but it could also be due to different legal frameworks. For example, Victoria has a long-standing, statutorily endorsed, and reasonably well known advance directive in the form of a Refusal of Treatment Certificate [24]. It may be that this is more entrenched among Victorian specialists than NSW advance directives which rely on the common law and have only been confirmed by Australian courts since 2009 [25]. More work is needed to understand variation between NSW and Victoria as this may shed light on how compliance with law can be enhanced.

These findings point to potential harms that non-compliance may have for patients, as well as for medical professionals who may be subject to civil actions, disciplinary hearings, and even criminal proceedings [11]. These findings also add weight to ongoing calls for law reform to achieve harmonisation in this area in Australia (and potentially in other countries as well). Securing compliance is a significant undertaking and all-the-more challenging when laws in different states or provinces within a country point in different directions. It is hard to justify why the three states surveyed need to have different legal frameworks for end-of-life decision-making. In the scenario considered in this survey, we consider that Queensland law should be changed so that the advance directive should be followed, in line with national (and generally international) trends. The ‘natural laboratory’ of a federation also provides an opportunity for harmonisation to select the best model. The empirical findings in this research, coupled with other medical, ethical and legal considerations, should be drawn upon to do that.

Finally, to the extent that lack of knowledge may lead to not following the law, these findings point to the need for more and better education. We have raised this elsewhere [11, 26] but this research suggests that education may be particularly important where the law does not match with what the profession regards as good medical practice. In other words, unusual law requires very specific knowledge, and particular attention should be paid to this in training and education so it is known and understood.

A limitation of this research is its response rate of 32%. While modest, this reflects the low and declining response rates detailed in other survey research involving doctors [27, 28]. While non-response bias cannot be excluded, it is reasonable to conclude that some non-responders are less likely to be interested in a survey about law and so would have less legal knowledge and less inclination to be influenced by the law than responders. If so, the potential bias in this study may actually overestimate the limited role for law that we found.

Further, survey respondents appear to be broadly representative of the wider sample from which they were drawn (Additional file 1: Comparison of AMPCo database and study sample). This sample included all doctors in the three largest Australian states who work in the seven specialities most likely to be involved in end-of-life decision-making. This sample is more representative of the profession than samples of earlier related studies looking at the legal knowledge and compliance of doctors in this area, which have generally been drawn from a single specialty or society [7, 9, 29–32], specific health facilities [10, 33–35] or specified training courses or cohorts [8, 36, 37]. However, the seven specialties used in the current sample are in the acute setting, which may limit the applicability of our results to other settings or other practitioners. Additionally, our findings regarding the role of law in Australia may not be generalisable to jurisdictions that lack comparable legal systems.

A further limitation of this study is that legal compliance was tested in a particular scenario and so does not assess the full spectrum of legal issues at the end of life [8]. The chosen scenario evoked a situation where medicine and law were in conflict. This was done to evaluate the impact of law on the respondents’ clinical decision-making; however different results may be obtained in a situation where it was not clinically challenging to follow the law. It is also acknowledged that the utility of advance directives in end-of-life care is contested [38], including because of challenges in interpretation, and these concerns may have coloured responses to the scenario.

## Conclusion

Despite purporting to regulate end-of-life care, it appears law may have a limited role in decision-making by doctors. This paper considered whether variation in law across Australian states would be reflected in different decisions but found that it was not. Instead, despite very different law in Queensland compared to NSW and Victoria, doctors from Queensland made broadly the same decision in the scenario and for broadly the same reasons as did doctors in NSW and Victoria. This suggests a need for more education about the law in this area, especially where the law may differ from what may be regarded as good medical practice. These findings also have implications for health policy-makers and legislators in terms of law reform, including reconsideration of the role and utility of law in guiding decisions about the provision of end-of-life care.

## Additional files

Additional file 1: Comparison of AMPCo database and study sample. This file compares the demographic data from the AMPCo database with that of the participants in the study sample. (PDF 87 kb)

Additional file 2: Explanation of Law: What does the Law Say? Responses by State. This file explains the legal position in relation to the survey question for the three Australian states being analysed in this paper. (PDF 87 kb)

## Abbreviation

NSW: New South Wales
