# Supplementary material for: Comparing doctors’ legal compliance across three Australian states for decisions whether to withhold or withdraw life-sustaining medical treatment: does different law lead to different decisions?
Source: BMC Palliat Care. 2017 Nov 28;16:63. doi: 10.1186/s12904-017-0249-1 (PMC5704501; doi:10.1186/s12904-017-0249-1)
Supplement: Supplementary file 1 — Comparison of AMPCo database and study sample. This file compares the demographic data from the AMPCo database with that of the participants in the study sample. (PDF 87 kb) [file 12904_2017_249_MOESM1_ESM.pdf]

Comparing doctors' legal compliance across three Australian states for decisions whether to withhold or withdraw life-sustaining medical treatment: does different law lead to different decisions?

Additional file 1

Professor Ben P White (Corresponding author)
Director, Australian Centre for Health Law Research, Faculty of Law,
Queensland University of Technology, Brisbane, Australia
bp.white@qut.edu.au
Ph: (+61) 7 3138 4066

Professor Lindy Willmott
Director, Australian Centre for Health Law Research, Faculty of Law,
Queensland University of Technology, Brisbane, Australia
l.willmott@qut.edu.au

Emeritus Professor Colleen Cartwright
Southern Cross University, Gold Coast, Australia
colleen.cartwright@scu.edu.au

Emeritus Professor Malcolm Parker
Faculty of Medicine,
University of Queensland, Brisbane, Australia,
m.parker@uq.edu.au

Professor Gail Williams
School of Public Health,
University of Queensland, Brisbane, Australia
g.williams@sph.uq.edu.au

Juliet Davis
Australian Centre for Health Law Research, Faculty of Law,
Queensland University of Technology, Brisbane, Australia
jed2169@columbia.edu

Comparison of AMPCo database and study sample

AMPCo provided marginal distributions by gender, age and main specialty for each state, based on the information in the AMPCo database for the selected specialties. The numbers of questionnaires sent to each specialty group were also recorded. We could not calculate exact response rates by each characteristic as responses could not be linked to the individual doctors selected. It is also possible that variable values differed in the AMPCo records and study questionnaire responses. Table S1 shows the marginal distributions of state, gender, age and specialty for the original sample from the AMPCo database and the study responses.

Comparison of AMPCo database and study sample by state, gender, age and main specialty

Characteristic		Total Surveyed<br>N = 2858<br>n (%)<br>AMPCo	Total Responses<br>N = 867<br>n (%)<br>Study
**State and Gender**			
Queensland	Males	461 (72)	148 (68)
	Females	175 (28)	69 (32)
NSW	Males	814 (66)	221 (66)
	Females	420 (34)	114 (34)
Victoria	Males	655 (66)	198 (63)
	Females	333 (34)	115 (37)
Missing gender		0	2
**Age**			
Less than 40 years		827 (29)	177 (20)
40 to 49 years		1047 (37)	336 (39)
50 to 59 years		568 (20)	219 (25)
60 years or older		266 (9)	117 (13)
Missing age		150 (5)	18 (2)
**Main Specialty**			
Emergency Medicine		1147 (40)	270 (31)
Geriatric Medicine		253 (9)	107 (12)
Intensive Care		428 (15)	125 (14)
Medical Oncology		338 (12)	80 (9)
Palliative Care		105 (4)	52 (6)
Renal Medicine		253 (9)	80 (9)
Respiratory Medicine		334 (12)	98 (11)
Missing specialty		0	55 (6)
