# Supplementary material for: Comparing doctors’ legal compliance across three Australian states for decisions whether to withhold or withdraw life-sustaining medical treatment: does different law lead to different decisions?
Source: BMC Palliat Care. 2017 Nov 28;16:63. doi: 10.1186/s12904-017-0249-1 (PMC5704501; doi:10.1186/s12904-017-0249-1)
Supplement: Supplementary file 2 — Explanation of Law: What does the Law Say? Responses by State. This file explains the legal position in relation to the survey question for the three Australian states being analysed in this paper. (PDF 87 kb) [file 12904_2017_249_MOESM2_ESM.pdf]

Comparing doctors' legal compliance across three Australian states for decisions whether to withhold or withdraw life-sustaining medical treatment: does different law lead to different decisions?

Additional file 2

Professor Ben P White (Corresponding author)
Director, Australian Centre for Health Law Research, Faculty of Law,
Queensland University of Technology, Brisbane, Australia
bp.white@qut.edu.au
Ph: (+61) 7 3138 4066

Professor Lindy Willmott
Director, Australian Centre for Health Law Research, Faculty of Law,
Queensland University of Technology, Brisbane, Australia
l.willmott@qut.edu.au

Emeritus Professor Colleen Cartwright
Southern Cross University, Gold Coast, Australia
colleen.cartwright@scu.edu.au

Emeritus Professor Malcolm Parker
Faculty of Medicine,
University of Queensland, Brisbane, Australia,
m.parker@uq.edu.au

Professor Gail Williams
School of Public Health,
University of Queensland, Brisbane, Australia
g.williams@sph.uq.edu.au

Juliet Davis
Australian Centre for Health Law Research, Faculty of Law,
Queensland University of Technology, Brisbane, Australia
jed2169@columbia.edu

Explanation of Law: What does the Law Say? Responses by State

NSW – The law says: do not commence antibiotics. This is because Mark has a valid advance care directive that applies to the situation that has arisen.

Victoria – The law says: do not commence antibiotics. Mark has a valid refusal of treatment certificate and his refusal of antibiotics is for a 'current condition' (his AIDS) that he had when completing this document. The position is not as certain as in NSW, however, due to the need to be refusing treatment for a current condition (which would have to be the AIDS rather than pneumonia).

Queensland – The law says: commence antibiotics. The conditions required for the advance health directive to apply under Queensland law (namely having a sufficiently serious medical condition and not having a reasonable prospect of regaining decision-making capacity) have not been met and the substitute decision-maker desires treatment.

Note: The law in these three jurisdictions is discussed in more detail in the below papers.

White B, Willmott L, Trowse P, et al. The legal role of medical professionals in decisions to withhold or withdraw life-sustaining treatment: part 1 (New South Wales). J Law Med 2011; 18: 498-522.

Willmott L, White B, Parker M, et al. The legal role of medical professionals in decisions to withhold or withdraw life-sustaining treatment: part 2 (Queensland). J Law Med 2011; 18: 523-544.

Willmott L, White, B, Parker M, et al. The legal role of medical professionals in decisions to withhold or withdraw life-sustaining treatment: part 3 (Victoria). J Law Med 2011; 18: 773-797.
